# Photosynthetic physiological characteristics, growth performance, and element concentrations reveal the calcicole–calcifuge behaviors of three *Camellia* species

**DOI:** 10.1515/biol-2022-0835

**Published:** 2024-03-09

**Authors:** Shengfeng Chai, Haidu Jiang, Yishan Yang, Xinfeng Pan, Rong Zou, Jianmin Tang, Zongyou Chen, Danjuan Zeng, Xiao Wei

**Affiliations:** Guangxi Key Laboratory of Functional Phytochemicals Research and Utilization, Guangxi Institute of Botany, Guangxi Zhuang Autonomous Region and Chinese Academy of Sciences, Guilin 541006, China

**Keywords:** yellow *Camellia* species, calcicole, calcifuge, acidic soils, calcareous soils, photosynthetic physiological characteristics, biomass, element concentrations

## Abstract

We grew three yellow *Camellia* species (the calcifuge *C. nitidissima* and *C. tunghinensis*, and the calcicole *C. pubipetala*) in acidic and calcareous soils for 7 months and assessed their photosynthetic physiological characteristics, growth performance, and element concentrations in this developmental context. The calcifuge *C. nitidissima* and *C. tunghinensis* species exhibited poor growth with leaf chlorosis, growth stagnation, and root disintegration in calcareous soils, and with their *P*_n_, *G*_s_, *T*_r_, *F*_v_/*F*_m_, ΦPSII, ETR, qP, leaf Chla, Chlb, and Chl(a + b) concentrations, and root, stem, leaf, and total biomass being significantly lower when grown in calcareous soils relative to in acidic soils. In contrast, the calcicole *C. pubipetala* grew well in both acidic and calcareous soils, with few differences in the above parameters between these two soil substrates. The absorption and/or transportation of nutrient elements such as N, K, Ca, Mg, and Fe by the two calcifuge *Camellia* species plants grown in calcareous soils were restrained. Soil type plays a major role in the failure of the two calcifuge *Camellia* species to establish themselves in calcareous soils, whereas other factors such as competition and human activity are likely more important limiting factors in the reverse case. This study furthers our understanding of the factors influencing the distribution of these rare and endangered yellow *Camellia* species, allowing for improved management of these species in conservation projects and horticultural production.

## 1 Introduction

Edaphic properties are key environmental factors that affect species diversity, distribution, and plant nutrition [1–4]. Calcareous soils and acidic soils are both widely distributed at the global level and are associated with distinct soil properties that are linked to consequent differences in species diversity. Calcareous soils are rich in Ca and has a higher pH and \[{\text{HCO}}_{3}^{-}]\] levels; moreover, the availability of phosphorus (P), iron (Fe), manganese (Mn), and zinc (Zn) is very low due to higher pH in this soil type [5–7]. In contrast, acidic soils have lower Ca and pH levels and higher Al concentrations [8]. Ecologists have classified plant species into calcifuges, which grow in acidic soils with low Ca^2+^ concentrations, and calcicoles, which grow in calcareous substrates [9]. Calcicole plants can grow well in calcareous soils, and can acclimate to the high calcium environment through a series of morphological and/or physiological functional adjustments [10], such as the synthesis of calcium oxalate crystals and storage thereof in tissues and intercellular spaces [11–13], the formation of calcified roots, depositing calcium [14], reducing root-mediated calcium absorption [15,16], increasing root organic acid secretion [17,18], or forming mycorrhizal associations that can promote the absorption of certain insoluble elements [19–21]. In contrast, calcifuge plants are usually sensitive to high calcium environments, and they grow poorly in calcareous soils primarily because of Ca-associated toxicity [22,23] and/or element deficiencies [24,25]. Some studies of the adaptability and their mechanistic basis of calcicole and calcifuge plants to acidic and calcareous soils have been conducted, but these experiments have primarily focused on single species or on different families and genera, with few such studies of calcicole–calcifuge behaviors among different species in the same genus having been conducted [26–28], especially for endangered plants.

*Camellia*, sect. *Chrysantha* Chang is an evergreen shrub or small tree in the Theaceae family that is renowned for its yellow camellia flowers, representing a rare ornamental plant group and germplasm resource of value for the breeding of new hybrid camellia varieties [29]. These plants primarily grow in Guangxi Province, South China and North Vietnam in tropical limestone evergreen broad-leaved forests, limestone mountain seasonal rainforests, and subtropical evergreen broad-leaved forests [30]. Plants in this group are most common in mountain valleys, alongside streams, and on limestone slopes from 120 to 350 m above sea level [31], and most have a narrow distribution and are listed in the list of rare and endangered plants in China. To date, 16 species that grow best in calcareous soils in limestone mountain regions have been described, while seven species that grow most readily in acidic soils in sandstone and shale mountains have been identified [32,33]. Under natural conditions, there have been no reports of the same species growing in both of these soil types [30]. *Camellia*, sect. *Chrysantha* Chang species can be separated into calcicole and calcifuge categories based upon their calcium dependence and whether they grow most readily in acidic or calcareous soils, respectively. These contrasting soil type preferences within the same genus are interesting but are not well understood.

Most calcicole yellow *Camellia* species can grow in acidic soils [34], but the adaptability of calcifuge *Camellia* species to calcareous soils remains unclear. The objectives of this study were to compare the performance of these three yellow *Camellia* species in these two soil types. We hypothesized that (1) the calcifuge yellow *Camellia* species would grow poorly and exhibit lower chlorophyll content and photosynthetic capacity in calcareous soils owing to an inability to acclimate to such soils owing to Ca-associated toxicity and/or element deficiencies and (2) the growth performance, chlorophyll content, and photosynthetic capacity of calcicole *Camellia* species in calcareous soils would be better or similar to those observed in acidic soils.

## 2 Materials and methods

### 2.1 Study site

The experiment was carried out in Guilin Botanical Garden, Yanshan District, Guilin City, Guangxi Province, China in 2018. This site is located at 25°11.2′N, 110°12.0′E at an altitude of 175 m in a subtropical monsoon climate zone with an average annual temperature of 19.2°C, average temperatures during the hottest and coldest months are 28.4 and 7.7°C, respectively, and with temperature extremes of 39 and –2°C. The average annual rainfall at this study site is 1854.8 mm, with most rain (73%) falling between April and August. On average, this site receives 1,680 h of sunshine per year, and the annual average relative humidity is 82%. Relative to the climate conditions experienced by the three species in their natural environments, the experimental site was to the north and the temperature was lower in the winter, but the three plants were still able to grow normally in acidic soil.

### 2.2 Treatments

Two calcifuge species (*Camellia nitidissima* and *Camellia tunghinensis*) and one calcicole species (*Camellia pubipetala*) were used in this experiment. The *C. nitidissima* plants used in this study were 1-year-old seedlings propagated from seeds collected from wild populations in Golden Camellia National Nature Reserve, Fangcheng, Guangxi (21°45.0′N, 108°5.9′E). This Reserve belongs to the north tropical monsoon climate, with an average annual temperature of 21.9°C, average temperature of the coldest month (January) is 12.6°C, extreme low temperature of −0.9°C, average temperature of the hottest month (July) is 28.3°C, and extreme high temperature of 39.1°C. The average sunshine hours per year is 1,525 h and the average annual rainfall is more than 2,500 mm. The *C. tunghinensis* plants used in this study were 2-year-old grafted seedlings propagated from the branches of wild plants in Golden Camellia National Nature Reserve. The *C. pubipetala* plants were 2-year-old grafted seedlings propagated from the branches of wild plants in Pingshan town, Long’an County, Guangxi (23°0.1′N, 107°34.8′E). This site has a subtropical monsoon climate, with hot summer and warm winter, abundant rainfall and comparatively high relative humidity. The average temperatures are 21.8°C as the annual mean, 13.2°C in January (the coldest month), and 33.2°C in July (the hottest month). Most of the rainfall is observed in the summer and autumn, with an annual mean of 1,500 mm. The original soils in which the two calcifuge species grown were acidic with low levels of calcium, while those of calcicole species were neutral or weakly alkaline with a high calcium content [35]. Before the experiment, the seedlings of these three species were planted in plastic bags containing loess and peat soil, with a volume ratio of 2:1, and all these seedlings are growing well.

Acidic and calcareous soils were used for this study. Acidic soils were collected from Guilin Botanical Garden. These were acidic, sticky, yellow soils with low levels of organic matter that were formed via the weathering of sandy shale, and were collected at a depth of 0–20 cm. The calcareous soils were collected from the crevices of karst hills in Gongcheng County, Guilin City, Guangxi Province at a collection depth of 0–20 cm. Plant residues and stones were removed after collection, and large pieces of soils were crushed and screened prior to use. Relative to the acidic soils, the calcareous soils exhibited a nearly neutral pH, higher levels of organic matter, total N, available Ca, and available Mg, and lower levels of available P (Table 1).

**Table 1:** Nutrient status of the soils used in this study

Soil type	pH value	Organic matter (%)	Total N (g/kg)	Total P (g/kg)	Total K (g/kg)	Available Ca (cmol(1/2Ca^2+^)/kg)	Available Mg (cmol(1/2Mg^2+^)/kg)
Acidic soils	5.34	1.26	1.53	1.09	14.11	2.05	0.18
Calcareous soils	7.07	14.29	14.63	2.05	11.12	21.60	2.34

In early April 2018, healthy plants with comparable levels of growth were selected to conduct pot-based experiments using acidic and calcareous soils as substrates. The roots of members of these three yellow *Camellia* species were washed and planted in plastic pots with an inner diameter of 30 cm and a depth of 25 cm. One plant was planted per pot, with 20 plants per experimental treatment. These three yellow *Camellia* species are shade-adapted plants, *C. nitidissima* and *C. pubipetala* grow best under 10% sunlight (among 10, 30, 50, and 100% of full sunlight) [36,37], and the light transmittance under forest canopy in the original habitat of these three species are less than 25% [38]. Therefore, in this experiment, we placed the potted plants in a shade shed with a relative light intensity of 10%. Regular weeding and pest control were conducted, but no fertilizer was applied during the experimental period to compare the effects of different soil types on seedling growth.

### 2.3 Sample collection and analysis

In early November 2018, the third to fifth mature functional leaves from the top of experimental plants were assessed to measure gas exchange parameters, chlorophyll fluorescence, and photosynthetic pigments. The biomass of the plants and the root and leaf nutrient concentrations therein were also measured.

#### 2.3.1 Determination of gas exchange parameters

The net photosynthetic rate (*P*_n_, μmol m^−2^ s^−1^), transpiration rate (*T*_r_, mol m^−2^ s^−1^), stomatal conductance (*G*_s_, mmol m^−2^ s^−1^), and intercellular CO_2_ concentration (*C*_i_, μmol mol^−1^) were assessed with a Li-6400 portable photosynthesis analyzer system (LI-COR, NE, USA). Measurements were made from 9:00 to 11:00 on sunny days. The photosynthetically active radiation was set at 300 μmol m^−2^ s^−1^. The maximum photon flux density experienced on a sunny day is about 2,000–2,500 μmol m^−2^ s^−1^ at the experimental site and it shall not exceed 300 μmol m^−2^ s^−1^ in the shade net. These three yellow *Camellia* species are shade-adapted plants, and the light saturation point of them are all less than 500 μmol m^−2^ s^−1^ [38,39]. Therefore, we chose 300 μmol m^−2^ s^−1^ as the measured light intensity for this study. The temperature of the control chamber was (28 ± 1)°C, the CO_2_ concentration in the sample chamber was (400 ± 5) mmol mol^−1^, the mean relative humidity was 53 ± 2%, and the flow rate was set at 500 µmol s^–1^.

#### 2.3.2 Chlorophyll fluorescence analyses

Chlorophyll fluorescence parameters of leaves were assessed with a Mini-Imaging-PAM modulation chlorophyll fluorescence imaging system (Walz Company, Germany) after 20 min of dark adaptation in the morning. The initial fluorescence (*F*_o_) was analyzed using a measuring light (0.1 μmol m^−2^ s^−1^), and then the maximum fluorescence (*F*_m_) was generated via excitation with a saturated 6,000 μmol m^−2^ s^−1^ light pulse (pulse time: 0.8 s). The minimum fluorescence ( \[{F}_{\text{o}}^{^{\prime} }]\] ), maximum fluorescence ( \[{F}_{\text{m}}^{^{\prime} }]\] ), and stable fluorescence (*F*_s_) of leaves under light adaptation were measured using a fluorescence kinetic curve induced by actinic light (55 μmol m^−2^ s^−1^), and the maximum photochemical efficiency (*F*_v_/*F*_m_), actual photochemical efficiency (ΦPSII), photosynthetic electron transfer rate (ETR), photochemical quenching (qP), and non-photochemical quenching (NPQ) were calculated with the Wincontrol-3 software [40].

#### 2.3.3 Determination of photosynthetic pigments

Leaf chlorophyll (Chl) and carotenoid (Car) contents were determined as per the methods of Lichtenthaler [41]. These pigments were extracted with 95% ethanol, and the absorbance of extracted liquids was recorded with a spectrophotometer (TU1901, Beijing Purkinje General Instrument Co., Ltd, China) at 665 and 649 nm for Chl, and at 470 nm for Car. The following formulae were then used to calculate pigment concentrations: Chla = 13.95 *A*_665_−6.88 *A*_649_, Chlb = 24.96 *A*_649_−7.32 *A*_665_, Car = (1,000 *A*_470_−2.05 Chla−114.8 Chlb)/245, as well as the ratios of Chla/b and Car/Chl.

#### 2.3.4 Biomass measurement

After 7 months of in-pot growth during the experimental period, whole plants were harvested and taken back to the laboratory for washing and drying. Plants were dried at 80°C to a constant weight in an oven, after which the stem, root, and leaf weights were determined with an electronic balance, and the biomass of each of these tissue types was calculated, as was total biomass.

#### 2.3.5 Nutrient element measurement

After biomass measurements were completed, root and leaf samples were ground and homogenized. The N, P, K, Ca, Mg, Fe, and Mn concentrations in these roots and leaves were then quantified. N concentrations were measured using the Kjeldahl method, with samples being digested using K_2_SO_4_:CuSO_4_·5H_2_O:Se (10:1:0.1) and H_2_SO_4_ [42]. Other nutrient (P, K, Ca, Mg, Fe, and Mn) levels were assessed by digesting samples using concentrated HNO_3_ [43], and were then analyzed using an inductively coupled plasma emission spectrum (iCAP Qc, Thermo Fisher Scientific, Bremen, Germany).

#### 2.3.6 Data processing

For the same species grown in different substrates, *t*-tests were used to assess the effects of soil type on gas exchange parameters, chlorophyll fluorescence, photosynthetic pigment content, biomass, and leaf/root nutrient element concentrations. SPSS 20.0 (SPSS Inc., IL, USA) was used for all statistical testing. Sigmaplot 12.5 was used to plot the resultant data (Systat Software, CA, USA).

## 3 Results

### 3.1 Gas exchange parameters

Relative to plants grown in acidic soils, the *P*_n_, *G*_s_, and *T*_r_ of the two calcifuge *Camellia* species grown in calcareous soils were significantly decreased (*P* < 0.01), while *C*_i_ was significantly increased (*P* < 0.05). The *P*_n_, *G*_s_, and *T*_r_ of *C. nitidissima* plants grown in calcareous soils were 9.51, 26.47, and 23.40% of those for plants grown in acidic soils, respectively, while for *C. tunghinensis* these respective values were 7.76, 9.84, and 8.70% (Table 2). The *C*_i_ of these two plants was 1.46 and 1.24 times higher, respectively, relative to corresponding plants grown in acidic soils. The *P*_n_ of calcicole *C. pubipetala* in calcareous soils was significantly higher than that in acidic soils (*P* < 0.05), while there was no significant difference in *G*_s_, *T*_r_, or *C*_i_ between the plants grown in these two soil types (*P* > 0.05) (Table 2).

**Table 2:** Photosynthetic gas exchange parameters of three yellow *Camellia* species grown in different soil types

Species	Soil type	*P*_n_ (μmol m^−2^ s^−1^)	*G*_s_ (mol m^−2^ s^−1^)	*T*_r_ (mmol m^−2^ s^−1^)	*C*_i_ (μmol mol^−1^)
*C. nitidissima*	Acidic soils	2.84 ± 0.61	0.034 ± 0.011	0.94 ± 0.24	245.08 ± 20.27
	Calcareous soils	0.27 ± 0.12	0.009 ± 0.001	0.22 ± 0.02	356.96 ± 17.22
		**	**	**	*
*C. tunghinensis*	Acidic soils	4.64 ± 0.49	0.061 ± 0.011	1.61 ± 0.31	259.33 ± 10.28
	Calcareous soils	0.36 ± 0.29	0.006 ± 0.002	0.14 ± 0.06	321.40 ± 36.30
		**	**	**	*
*C. pubipetala*	Acidic soils	3.09 ± 0.45	0.054 ± 0.011	1.01 ± 0.357	297.39 ± 7.37
	Calcareous soils	3.66 ± 0.47	0.055 ± 0.010	0.95 ± 0.20	281.04 ± 18.19
		*	ns	ns	ns

Data are mean ± SD (*n* = 6). Significance (*t*-test): *P < 0.05, **P < 0.01; ns: not significant.

### 3.2 Chlorophyll fluorescence

Relative to those of plants grown in acidic soils, the *F*_v_/*F*_m_, ΦPSII, ETR, and qP of the two calcifuge *Camellia* species grown in calcareous soils were significantly decreased (*P* < 0.05), while NPQ were significantly increased (*P* < 0.01) (Table 3). The chlorophyll fluorescence parameters of the calcicole *C. pubipetala* did not exhibit any significant differences between acidic and calcareous soils (*P* > 0.05).

**Table 3:** Chlorophyll fluorescence parameters for three yellow *Camellia* species grown in different soil substrates

Species	Soil type	*F*_v_/*F*_m_	*Φ*PSII	ETR	qP	NPQ
*C. nitidissima*	Acidic soils	0.79 ± 0.01	0.63 ± 0.04	14.53 ± 0.88	0.88 ± 0.04	0.52 ± 0.13
	Calcareous soils	0.72 ± 0.03	0.39 ± 0.08	8.50 ± 1.71	0.75 ± 0.14	2.21 ± 0.45
		*	*	*	*	**
*C. tunghinensis*	Acidic soils	0.79 ± 0.01	0.65 ± 0.03	15.06 ± 0.80	0.91 ± 0.02	0.47 ± 0.16
	Calcareous soils	0.69 ± 0.03	0.42 ± 0.05	9.78 ± 1.12	0.87 ± 0.01	1.54 ± 0.25
		*	*	*	*	**
*C. pubipetala*	Acidic soils	0.79 ± 0.01	0.60 ± 0.02	13.86 ± 0.53	0.86 ± 0.03	0.62 ± 0.10
	Calcareous soils	0.80 ± 0.01	0.59 ± 0.06	15.85 ± 4.11	0.83 ± 0.06	0.63 ± 0.19
		ns	ns	ns	ns	ns

Data are mean ± SD (*n* = 6). Significance (*t*-test): *P < 0.05, **P < 0.01; ns: not significant.

### 3.3 Photosynthetic pigment contents and ratios

The Chla, Chlb, Chl(a + b), and Car contents of the two calcifuge *Camellia* species grown in calcareous soils were significantly lower than those of plants grown in acidic soils (*P* < 0.05 or 0.01), while the Chla/b and Car/Chl values were significantly higher than those in acidic soils (*P* < 0.05) (Table 4). There was no significant difference in these parameters when comparing calcicole *C. pubipetala* plants grown in acidic and calcareous soils (*P* > 0.05).

**Table 4:** Photosynthetic pigment contents and ratios in the leaves of three yellow *Camellia* species grown in different soil substrates

Species	Soil type	Chla (mg g^−1^ FW)	Chlb (mg g^−1^ FW)	Chl(a + b) (mg g^−1^ FW)	Car (mg g^−1^ FW)	Cha/b	Car/Chl
*C. nitidissima*	Acidic soils	1.66 ± 0.08	0.66 ± 0.03	2.32 ± 0.10	0.26 ± 0.05	2.50 ± 0.14	0.11 ± 0.02
	Calcareous soils	0.87 ± 0.09	0.39 ± 0.05	1.26 ± 0.10	0.22 ± 0.03	3.27 ± 0.31	0.18 ± 0.02
		**	**	**	*	*	*
*C. tunghinensis*	Acidic soils	2.09 ± 0.25	0.53 ± 0.06	2.62 ± 0.35	0.55 ± 0.08	3.92 ± 0.13	0.21 ± 0.01
	Calcareous soils	1.17 ± 0.08	0.25 ± 0.03	1.42 ± 0.10	0.36 ± 0.02	4.67 ± 0.51	0.25 ± 0.03
		**	**	**	*	*	*
*C. pubipetala*	Acidic soils	1.92 ± 0.11	0.81 ± 0.05	2.83 ± 0.16	0.33 ± 0.02	2.38 ± 0.02	0.12 ± 0.01
	Calcareous soils	1.85 ± 0.10	0.78 ± 0.05	2.63 ± 0.15	0.30 ± 0.01	2.39 ± 0.03	0.11 ± 0.01
		ns	ns	ns	ns	ns	ns

Data are mean ± SD (*n* = 4). Significance (*t*-test): *P < 0.05, **P < 0.01; ns: not significant.

### 3.4 Plant appearance and biomass

**Figure 1:**
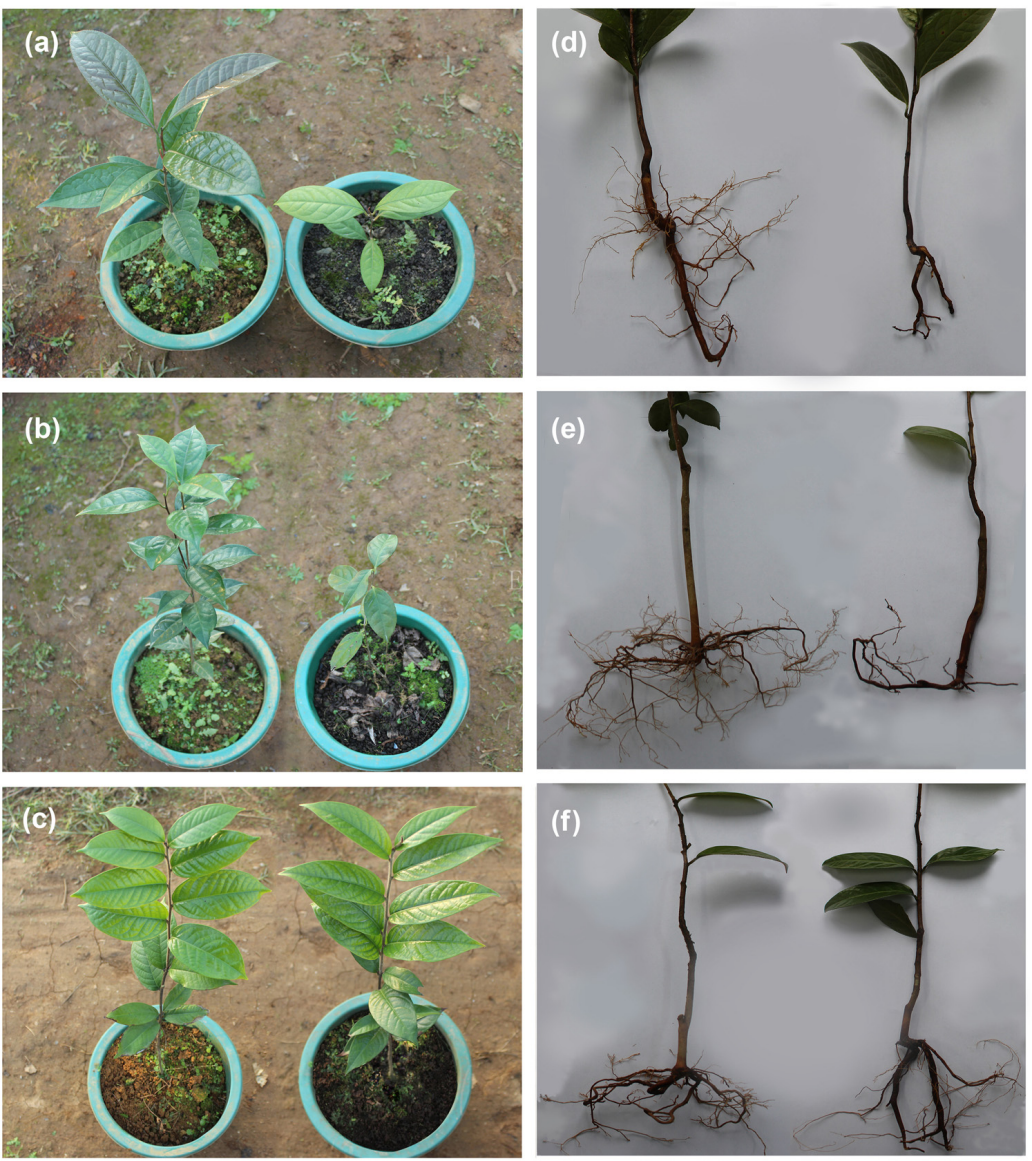
Growth performance of three yellow *Camellia* species grown in different soil substrates: (a, d) *C. nitidissima*, (b, e) *C. tunghinensis*, (c, f) *C. pubipetala*. The plants grown in acidic and calcareous soils are shown on the left and right, respectively.

Almost no growth was observed for either of these two calcifuge *Camellia* species in calcareous soils, and they exhibited leaf chlorosis, some of the leaves wilting and loss, root disintegration. The calcicole *C. pubipetala* plants grew well in both soils (Figure 1). The root biomass, stem biomass, leaf biomass, and total biomass of the two calcifuge *Camellia* species grown in calcareous soils were significantly lower than those of plants grown in acidic soils (*P* < 0.01) (Table 5). The root, stem, leaf and total biomass of *C. nitidissima* grown in calcareous soils were 40.85, 51.81, 33.23, and 40.10% of those grown in acidic soils, respectively, while for *C. tunghinensis* these respective values were 38.79, 55.65, 36.84, and 43.61% (Table 5). The root and stem biomass of the calcicole *C. pubipetala* grown in calcareous soils were similar to those of plants grown in acidic soils, and the leaf biomass and total biomass of these plants were higher than those grown in acidic soils, although the difference was not significant (*P* > 0.05).

**Table 5:** Biomass of three yellow *Camellia* species grown in different soil substrates

Species	Soil type	Root biomass (g)	Stem biomass (g)	Leaf biomass (g)	Total biomass(g)
*C. nitidissima*	Acidic soils	1.42 ± 0.15	1.66 ± 0.17	3.19 ± 0.36	6.26 ± 0.34
	Calcareous soils	0.58 ± 0.10	0.86 ± 0.18	1.06 ± 0.17	2.51 ± 0.28
		**	**	**	**
*C. tunghinensis*	Acidic soils	2.32 ± 0.28	2.39 ± 0.29	2.66 ± 0.20	7.36 ± 0.38
	Calcareous soils	0.90 ± 0.18	1.33 ± 0.14	0.98 ± 0.10	3.21 ± 0.28
		**	**	**	**
*C. pubipetala*	Acidic soils	2.61 ± 0.44	2.31 ± 0.09	2.15 ± 0.30	7.07 ± 0.62
	Calcareous soils	2.78 ± 0.34	2.32 ± 0.18	2.63 ± 0.25	7.74 ± 0.70
		ns	ns	ns	ns

Data are mean ± SD (*n* = 4). Significance (*t*-test): *P < 0.05, **P < 0.01; ns: not significant.

### 3.5 Element concentrations

**Figure 2:**
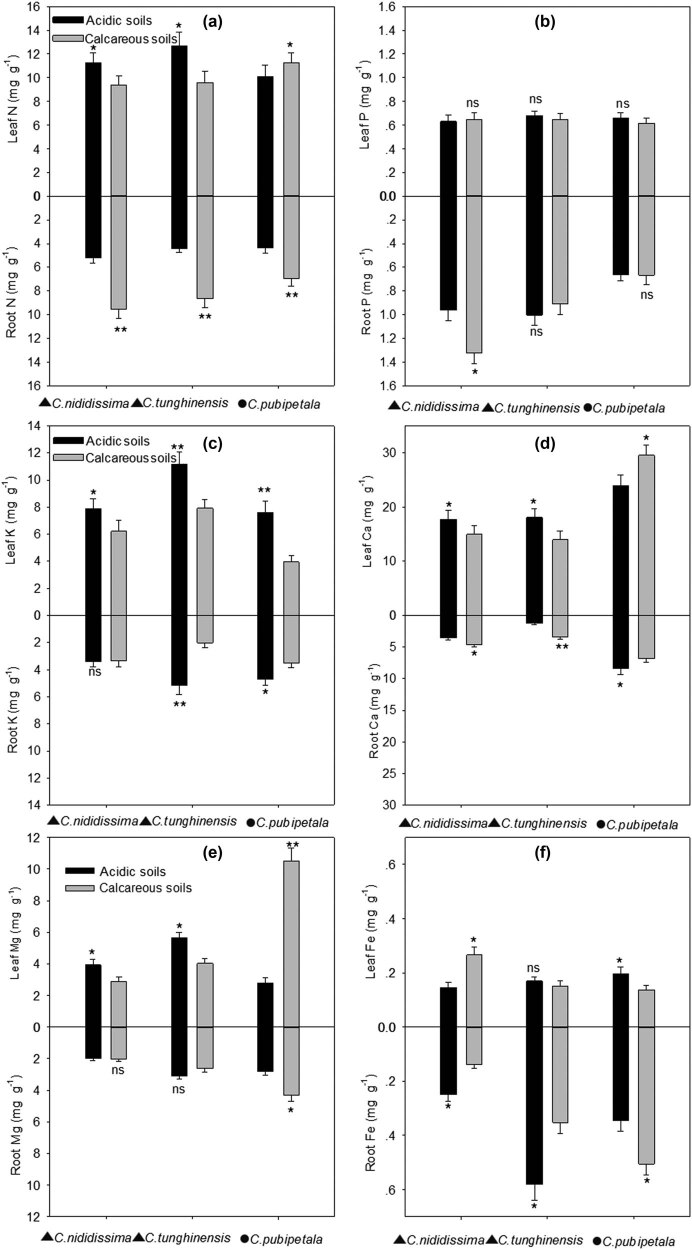
Leaf and root nutrient concentrations of three yellow *Camellia* species grown in different soil substrates. Data are mean ± SD (*n* = 3). Significance (*t*-test): *P < 0.05, **P < 0.01; ns: not significant. ▲ denotes calcifuge species, ● denotes calcicole species.

The leaf N concentrations of the two calcifuge *Camellia* species grown in calcareous soils were significantly lower than those of plants grown in acidic soils (*P* < 0.05), while the root N concentrations were significantly higher than those of plants grown in acidic soils (*P* < 0.01). The leaf and root N concentrations of the calcicole *C. pubipetala* plants grown in calcareous soils were higher than those of plants grown in acidic soils (Figure 2a) (*P* < 0.05 or 0.01). Aside from the root P concentrations in *C. nitidissima*, which were significantly higher for plants grown in calcareous soils relative to plants grown in acidic soils, there were no significant differences in leaf or root P concentrations for these three yellow *Camellias* species grown in different soil substrates (Figure 2b) (*P* > 0.05). Leaf and root K concentrations for three yellow *Camellia* species grown in calcareous soils were lower than those of plants grown in acidic soils (Figure 2c).

The leaf Ca concentrations of the calcifuge *Camellia* plants grown in calcareous soils were significantly lower than those of plants grown in acidic soils (*P* < 0.05), while root Ca concentrations were significantly higher relative to those in acidic soils (*P* < 0.05 or 0.01) (Figure 2d). The leaf Ca concentrations of the calcicole *C. pubipetala* were significantly higher in calcareous soils relative to acidic soils, whereas the opposite was true for root Ca concentrations (*P* < 0.05).

Leaf Mg concentrations were significantly lower in calcifuge *Camellia* plants grown in calcareous soils relative to plants grown in acidic soils (*P* < 0.05), whereas there were no significant differences in root Mg concentrations between these two soil types (*P* > 0.05) (Figure 2e). The root and leaf Mg concentrations of the calcicole *C. pubipetala* in calcareous soils were significantly higher than those in acidic soils (*P* < 0.05 or 0.01).

Leaf Fe concentrations of the calcifuge *C. nitidissima* were significantly higher following growth in calcareous soils relative to acidic soils (*P* < 0.05), whereas no such differences were observed for calcifuge *C. tunghinensis* plants (*P* > 0.05) (Figure 2f). Root Fe concentrations in these two calcifuge *Camellia* species were significantly lower following growth in calcareous soils relative to acidic soils (*P* < 0.05). Leaf Fe concentrations in calcicole *C. pubipetala* plants were significantly lower following growth in calcareous soils relative to acidic soils, whereas the opposite was observed in the roots of these plants (*P* < 0.05).

With the exception of *C. nitidissima*, which exhibited no difference in leaf Mn concentrations when grown in these two soil types, the leaf and root Mn concentrations in these three yellow *Camellia* species were significantly lower following growth in calcareous soils relative to acidic soils (*P* < 0.05) (Figure S1).

## 4 Discussion

Calcifuge plants usually grow poorly in calcareous soils due to Ca-associated toxicity and/or element deficiencies, which primarily manifest in the form of leaf chlorosis or necrosis, decreased chlorophyll content, and reductions in photosynthetic capacity and biomass [27,28,44,45]. In contrast, calcicole plants are usually insensitive to soil calcium content, and most grow normally in both acidic and calcareous soils [44,46]. This study was the first to focus on the calcicole–calcifuge behaviors of *Camellia* species, and attempts to elucidate the endangered mechanism of yellow *Camellia* species from the perspective of soil factors. In this study, when grown in calcareous soils the two calcifuge *Camellia* species showed reduced photosynthetic capacity, growth stagnation, root disintegration, nutrient deficiency, and negative impacts on physiology, while the calcicole *C. pubipetala* plants can grow well, and allocate more Ca to their leaves compared with calcifuges. The results of this study are partly similar to those of previous studies, but can also provide some new insight into the mechanism driving these patterns of growth.

In this experiment, the *P*_n_, *G*_s_, and *T*_r_ of the two calcifuge *Camellia* species were significantly lower when grown in calcareous soils relative to acidic soils, and their *P*_n_ values were less than 10% of the values observed following growth in acidic soils. This indicates that these two calcifuge *Camellia* species were under severe stress in calcareous soils, consistent with their leaf chlorosis and growth stagnation. The decrease in *P*_n_ was accompanied by an increase in *C*_i_, indicating that the former was mainly caused by non-stomatal factors [47], such as impaired carboxylation ability or decreased chlorophyll content in mesophyll cells [48]. This was consistent with the significant decrease of chlorophyll fluorescence parameters ETR, *Φ*PSII, qP, and chlorophyll content of the calcifuge *Camellia* species grown in calcareous soils, and these manifestations may be related to element deficiencies and Ca-associated toxicity. There was little difference in photosynthetic capacity, chlorophyll fluorescence parameters, and chlorophyll content of the calcicole *C. pubipetala* plants when grown in these two soil types, suggesting that *C. pubipetala* was readily able to acclimate to both of these soil substrates.

Under normal physiological conditions, the *F*_v_/*F*_m_ values of the vast majority of C_3_ plants are in the 0.8–0.84 range [49]. If this value is greatly reduced, it indicates that the plant is subject to environmental stress [50]. In this experiment, the *F*_v_/*F*_m_ values of the two calcifuge *Camellia* species grown in calcareous soils were 0.72 and 0.69, respectively, which may be attribute to the down regulation of photosynthetic function, although the damage of photosynthetic structure cannot be ruled out. Changes in the Chla/b ratio are related to the balance of light absorption capacity of photosystems [51]. The Car/Chl ratio reflects the relationship between light absorption and protection against light damage protection in plants [52]. In this experiment, the higher Chla/b and Car/Chl ratios of the two calcifuge *Camellia* species grown in calcareous soils were able to reduce the absorption of light energy by increasing heat dissipation, and this may represent a protective mechanism whereby plants can cope with stress conditions.

Plant biomass accumulation is an indicator of net carbon gain. Calcifuge species generally grow poorly and exhibit lower biomass in calcareous soils relative to acidic soils, while calcicole species present with lower biomass in acidic soils than in calcareous soils [28], or with no significant difference between these two soil types [44,53]. In this experiment, the root biomass, stem biomass, leaf biomass, and total biomass of calcifuge plants grown in calcareous soils were significantly less than those of plants grown in acidic soils, especially for roots, calcifuge plants grown in calcareous soils had almost no fibrous roots. In contrast, no significant difference in biomass was observed when calcicole *C. pubipetala* plants were grown in acidic or calcareous soils. This suggests that soil type plays a major role in the failure of the two calcifuge *Camellia* species to establish themselves in calcareous soils, whereas other factors such as competition and human activity are likely more important limiting factors in the reverse case [44,54]. The low fruit and seed set of *C. pubipetala* cultivated in acidic soils (personal data) should make it weak in community competition, which may be an important factor for limiting the distribution of this species in acidic soils. Although there are soil hills in acidic soils near the native population sites of *C. pubipetala*, the habitat suitable for the growth of this species has been destroyed by human activities, which may be another reason for excluding this species from acidic soils.

In calcareous soils, phosphate availability in plants is reduced owing to the fact that high pH values and Ca^2+^ concentrations can result in Ca phosphate precipitation and apatite formation [55]. P deficiency is known to impair calcifuge plant growth in calcareous soils [56–58], and such impairment is believed to be related to the reduced ability of these plants to solubilize phosphate relative to calcicole species [14,59,60]. In this experiment, there were no significant differences in leaf P concentrations between the two calcifuge *Camellia* species grown in different soil substrates, and the root P concentration of *C. nitidissima* in calcareous soils was even higher than that in acidic soils, this result is not consistent with the significant decrease of P concentrations in leaves of some calcifuge plants grown in calcareous soils [57], indicating that P deficiency may not be the reason for the inability of these two calcifuge *Camellia* species to grow in calcareous soils.

High pH values and \[{\text{HCO}}_{3}^{-}]\] concentrations cause Fe deficiency chlorosis in plants grown in calcareous soils [61], both by reducing soil Fe solubility and by inhibiting Fe uptake, metabolism, and translocation [62–64]. Therefore, synthetic and natural chelators are widely used in cropping systems to improve iron and other micro-nutrients in plants [65]. Although the leaf Fe concentrations in the two calcifuge plants were not significantly lower when grown in calcareous soils than when grown in acidic soils, the change of biologically available Fe concentration in leaves was not clear, so whether there was Fe deficiency in calcifuge *Camellia* plants grown in calcareous soils still needs further verification.

Calcium toxicity is one of the major causes of calcifuge plant sensitivity to calcareous soils [66–68]. High Ca levels may disturb the lamellar structure of chloroplasts and consequently decrease net photosynthetic rates, and high Ca availability in the rhizosphere can also reduce cell wall extensibility, leaf expansion rate, and root elongation [69–71]. Excessive Ca may precipitate with P as Ca_3_(PO_4_)_2_ in plant tissues, thereby rendering both Ca and P unavailable [72–74]. In this experiment, the significantly elevated root Ca concentrations and decreased leaf Ca concentrations of the two calcifuge *Camellia* species grown in calcareous soils relative to acidic soils, combined with significantly reduced root biomass and root disintegration, indicating that the roots, rather than the leaves, of these two calcifuge *Camellia* species grown in calcareous soils may suffer toxic effects of exposure to high calcium levels. This was partially confirmed by another experiment. Under high calcium treatment, the photosynthetic capacity, chlorophyll fluorescence parameters ΦPSII, ETR, and chlorophyll content of the two calcifuge *Camellia* species decreased significantly [75]. This result is in contrast to findings in some calcifuge plants grown in high calcium environments, which exhibit higher root and leaf calcium concentrations [27,28,67]. Calcicole species tend to exhibit tight control over Ca uptake and/or translocation from roots to leaves, or to achieve better Ca compartmentation at the cellular level [73], these abilities likely play an important role in the tolerance of some calcicole species to calcareous soils [76–78]. The leaf Ca concentrations of calcicole *C. pubipetala* plants grown in calcareous soils were significantly higher than those of plants grown in acidic soils, and they were also much higher than those of the calcifuge species grown in calcareous soils, indicating that this species exhibits a strong leaf Ca absorption and storage capacity when grown in high calcium environment [79]. This high Ca tolerance is most likely achieved through the biomineralization of excess Ca, leading to the formation of Ca-based minerals (presumably Ca oxalate), thereby avoiding any Ca^2+^ interference with cell functioning or with the availability/allocation of other nutrients [80,81]. The results indicated that the calcicole *C. pubipetala* is a high-Ca species [82].

The leaf N, Mg, and K concentrations of the two calcifuge *Camellia* species in calcareous soils were significantly lower than those in acidic soils, which may be attributable to the metabolic imbalance caused by the damage root.

## 5 Conclusions

The calcifuge *C. nitidissima* and *C. tunghinensis* plants exhibited poor growth in calcareous soils with leaf chlorosis, growth stagnation, and root disintegration. The *P*_n_, *G*_s_, *T*_r_, *F*_v_/*F*_m_, *Φ*PSII, ETR, qP, leaf Chl a, Chl b, and Chl(a + b) concentrations, as well as the root, stem, leaf, and total biomass of these two calcifuge *Camellia* species were significantly reduced when grown in calcareous soils relative to when grown in acidic soils. The absorption and/or transportation of the nutrient elements such as N, K, Ca, Mg, and Fe by the two calcifuge *Camellia* species plants grown in calcareous soils were restrained probably attributable to the damaged root. In contrast, the calcicole *C. pubipetala* grew well in both acidic and calcareous soils, with few differences in the photosynthetic parameters and growth performance between these two soil substrates. Soil type plays a major role in the failure of the two calcifuge *Camellia* species to establish themselves in calcareous soils, whereas other factors such as competition and human activity are likely more important limiting factors in the reverse case. This is the first study to demonstrate how calcareous soils impact yellow *Camellia* species, thus helping to explain why some yellow *Camellia* species are excluded from calcareous habitats. This study furthers our understanding of the factors influencing the distribution of these rare and endangered yellow *Camellia* species, allowing for improved management of these species in conservation projects and horticultural production.

## Supplementary Material

Supplementary Figure
