# Supplementary material for: Photosynthetic physiological characteristics, growth performance, and element concentrations reveal the calcicole–calcifuge behaviors of three Camellia species
Source: Open Life Sci. 2024 Mar 9;19(1):20220835. doi: 10.1515/biol-2022-0835 (PMC10997145; doi:10.1515/biol-2022-0835)
Supplement: Supplementary Figure [file biol-2022-0835-sm.pdf]

Supplementary material

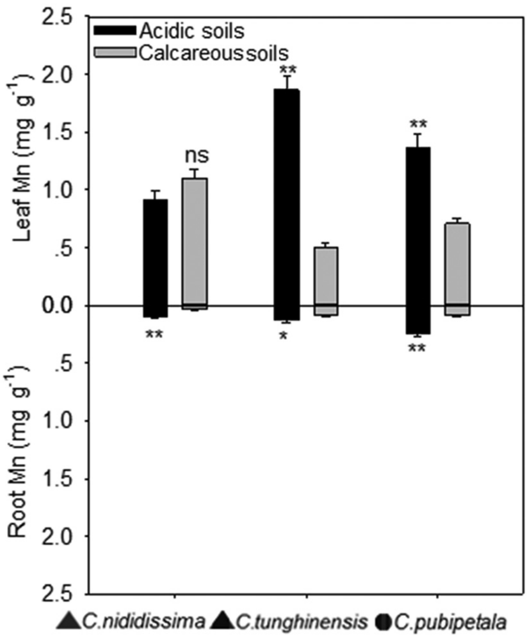

Figure S1: The leaf and root Mn concentrations of three yellow *Camellia* species grown in different soil substrates Data are means ± SD (n = 3). Significance (t-test): *P < 0.05, **P < 0.01; ns: Not significant. ▲ Denotes calcifuge species, ● denotes calcicole species.
